# Short- and long-term reproductive effects of prenatal and lactational growth restriction caused by maternal diabetes in male rats

**DOI:** 10.1186/1477-7827-9-154

**Published:** 2011-12-06

**Authors:** Elaine MP Amorim, Débora C Damasceno, Juliana E Perobelli, Raquel Spadotto, Carla DB Fernandez, Gustavo T Volpato, Wilma DG Kempinas

**Affiliations:** 1 Center of Biological and Health Sciences (CCBS), State University of West Paraná (UNIOESTE), Cascavel, Paraná, Brazil; 2 Department of Gynecology and Obstetrics, Botucatu Medical School, UNESP - Univ Estadual Paulista, Botucatu, São Paulo, Brazil; 3 Graduate Program in Cell and Structural Biology, Institute of Biology, University of Campinas, Campinas, São Paulo, Brazil; 4 Department of Morphology, Institute of Biosciences, UNESP - Univ Estadual Paulista, 18618-970, Botucatu, São Paulo, Brazil

**Keywords:** developmental programming, growth restriction, male rat, maternal diabetes, puberty, sexual development

## Abstract

**Background:**

A suboptimal intrauterine environment may have a detrimental effect on gonadal development and thereby increases the risk for reproductive disorders and infertility in adult life. Here, we used uncontrolled maternal diabetes as a model to provoke pre- and perinatal growth restriction and evaluate the sexual development of rat male offspring.

**Methods:**

Maternal diabetes was induced in the dams through administration of a single i.v. dose of 40 mg/kg streptozotocin, 7 days before mating. Female rats presenting glycemic levels above 200 mg/dL after the induction were selected for the experiment. The male offspring was analyzed at different phases of sexual development, i.e., peripuberty, postpuberty and adulthood.

**Results:**

Body weight and blood glucose levels of pups, on the third postnatal day, were lower in the offspring of diabetic dams compared to controls. Maternal diabetes also provoked delayed testicular descent and preputial separation. In the offspring of diabetic dams the weight of reproductive organs at 40, 60 and 90 days-old was lower, as well as sperm reserves and sperm transit time through the epididymis. However the plasma testosterone levels were not different among experimental groups.

**Conclusions:**

It is difficult to isolate the effects directly from diabetes and those from IUGR. Although the exposure to hyperglycemic environment during prenatal life and lactation delayed the onset of puberty in male rats, the IUGR, in the studied model, did not influenced the structural organization of the male gonads of the offspring at any point during sexual development. However the decrease in sperm reserves in epididymal cauda and the acceleration in sperm transit time in this portion of epididymis may lead to an impairment of sperm quality and fertility potential in these animals. Additional studies are needed in attempt to investigate the fertility of animals with intrauterine growth restriction by maternal diabetes and possible multigenerational effects.

## Background

Epidemiological studies and controlled experimental investigations in several species have demonstrated that impaired fetal growth is associated with long-term health effects [1-3]. In humans, a relationship between intrauterine growth restriction (IUGR) and cardiac diseases, hypertension, type 2 diabetes, resistance to insulin, and obesity has been well documented, making evident the role of prenatal programming as a determinant of diseases in the adult [4-7]. However, few studies have investigated the impact of the IUGR on reproductive function [8,9].

Fetal growth retardation seems to be associated with an increased risk of premature adrenarche, early puberty, polycystic ovary syndrome and associated fertility problems [10-13]. In males, prospectives studies show that impaired intrauterine growth increases the risk of congenital hypospadias, cryptorchidism and testicular cancer approximately two- to threefold [14-16]. Although few studies focus on the effect of intrauterine growth conditions on male sexual development and sperm quality later in life, previous studies suggested an impairment of both Sertoli and Leydig cell functions [8].

Different experimental models have been used to investigate the effects of IUGR in the offspring development: exposure of dams to isocaloric diet with low protein content [17-19]; global restriction of nutrient [20]; restriction of uterine blood flow [21,22]; exposure of fetuses to elevated levels of glycocorticoids [23] and experimental induction of maternal diabetes [24-26]. Animal models of maternal diabetes during pregnancy and/or lactation or neonatal overnutrition have provided valuable insight into mechanisms involved in perinatal programming of diseases in adult life. Furthermore, numerous animal experiments have been performed to understand the epidemiological association between IUGR and subsequent disease risk.

In the present study we examined the male offspring exposed to maternal diabetes during pregnancy and lactation to determine whether growth restriction, in this experimental model, disturbs pubertal and sexual development. Animal models are necessary to clarify pathogenetic and pathophysiologic aspects of IUGR. They also make it possible to investigate short and long term effects of untreated diabetics in pregnant rats, which is not ethically possible in humans [27].

## Methods

### Animals

Thirty male and 74 female Wistar rats aged 30 days were supplied by the Multidisciplinary Center for Biological Investigation of the University of Campinas, CEMIB - UNICAMP. The animals were adapted and maintained in the Small Mammal Biotherium of the Department of Morphology in the Institute of Biosciences at Botucatu, UNESP, where they were housed in collective polyethylene cages (43 × 30 × 15), under controlled conditions of temperature, maintained between 22° and 25°C, relative humidity of approximately 55% and 12 h photoperiod (light period beginning at 0700 h), with free access to water and food. At 90 days of age, the experimental period was initiated. The experimental protocol followed the Ethical Principles in Animal Experimentation adopted by the Brazilian College of Animal Experimentation (COBEA) and was approved by the Commission of Ethics in Animal Experimentation (CEEA) of the Institute of Biosciences at Botucatu (protocol 05/05).

### Experimental sequence for induction of diabetes in rats

At 90 days of age, diabetes was induced in 57 female rats using streptozotocin (STZ - SIGMA Chemical Company, St. Louis, MO, USA). STZ was administered i.v. into the tail vein in a single dose of 40 mg/kg dissolved in citrate buffer (0.1 M, pH 4.5). Control rats (n = 17) received (i.v.) citrate buffer. Blood glucose concentrations were measured 7 days after induction of diabetes. Glycemia was determined by collecting one drop of blood of the distal part of the rat tail and depositing it in glucose oxidase reagent strips, which were read in a specific glycosimeter (*One Touch Ultra *- Johnson & Johnson^®^). Only rats with blood glucose levels higher than 200 mg/dL were included in the diabetic group.

### Natural matings and obtainment of offspring

Eight days after administration of STZ, the estrous cycle was evaluated daily by vaginal smear and when females were in estrus phase, they were placed in the male cage and allowed to mate with non-diabetic male rats overnight (one female/male rat). In the next morning males and females were separated and gestational day zero (GD0) was determined by the presence of sperm in vaginal smears. After 15 days of mating procedure, the rats that did not present a positive pregnancy were discarded. Timed-pregnant female rats were housed individually and observed daily for delivery. For the indirect evaluation of the maternal toxicity, the rats were weighed in alternating days (from GD0 to 20), for control of weight gain. On the mornings of GD 0, 7, 14 and 21 blood samples were collected for determination of maternal glycemia following the procedures utilized during the diabetogenic period. The food and water intake were also measured in alternate days.

From GD20 pregnant rats were observed for delivery that was considered as day 0 of post natal life (PND0). All rats presented spontaneous vaginal delivery. Litters with less than 8 pups or litters that were cannibalized in part or totally were discharged. Pups were not weighed at birth to prevent maternal rejection [27]. At PND3 all litters were weighed and glycemic levels were determined. The number of pups per litter was reduced to eight when necessary [28], in order to maintain, preferentially, male offspring. Two groups were established: OC (offspring of control dams) and OD (offspring of diabetic dams).

### Evaluation of male offspring after birth

After weaning (PND 21) all pups were fed with control diet *ad libitum *and maintained in groups of up to four/cage until puberty. The litter was utilized as the sample unit for the parameters evaluated up to PND 39. Reproductive development was evaluated at the following ages: peripuberty (PND 40), postpuberty (PND 60) and adulthood (PND90), in groups of 6 to 8 rats each, one or two per litter. Body weight and glycemic levels of offspring were evaluated at PND3, 10, 40, 60 and 90.

### Evaluation of external physical signs of male sexual development (onset of puberty)

Starting on PND15 the time of testicular descent was determined by daily palpation of the scrotal sac. Preputial separation began to be investigated on PND33 through manual retraction of the prepuce.

### Collection of reproductive organs

At ages predicted, the animals were anesthetized with ethyl ether and killed by decapitation. Blood was collected from the ruptured cervical vessels for the determination of total testosterone concentration, as described below. The right testis and epididymis were removed and weighed, and in rats aged 60 and 90 days they were frozen at -4°C to be processed for counting of germ cells. The seminal vesicle (full, without the coagulating gland) and ventral prostate were also removed, weighed and discarded. At different ages of sexual development, the left testis and epididymis were removed and immersed in Alfac fixative mixture (85% alcohol 80°, 10% formaldehyde, and 5% acetic acid) for 24 h. The tissues were embedded in Paraplast, sectioned at 5 μm and stained with hematoxilin and eosin (HE) for evaluation of spermatogenesis and histopathological analysis.

### Daily sperm production per testis, sperm number and transit time in the epididymis

The sperm concentration was estimated in rats at PND60 and 90. Homogenization-resistant testicular spermatids (stage 19 of spermiogenesis) and sperm in the caput/corpus and cauda epididymidis were enumerated as described previously by Robb et al. [29], with adaptations described by Fernandes et al.[30]: the right testis, decapsulated and weighed soon after collection, were homogenized in 5 ml of NaCl 0.9% containing Triton × 100 0.5%, followed by sonication for 30 sec. After a 10-fold dilution a sample was transferred to Newbauer chambers (4 fields per animal), preceding a count of mature spermatids. To calculate daily sperm production (DSP) the number of spermatids at stage 19 was divided by 6.1, which is the number of days of the seminiferous cycle in which these spermatids are present in the seminiferous epithelium. In the same manner, caput/corpus and cauda epididymidis portions were cut into small fragments with scissors and homogenized, and sperm counted as described for the testis. The sperm transit time through the epididymis was determined by dividing the number of sperm in each portion (caput/corpus and cauda) by DSP.

### Sperm morphology

For the evaluation of sperm morphology, the left vas deferens of 90-day-old rats was sectioned at its extremities and washed with the aid of a syringe coupled to a needle, containing 1.0 mL of formol-saline solution. The washed product was collected in an Eppendorf tube and, soon after, smears were prepared in histological slides and left to dry in open air. With the aid of a phase-contrast microscope, the smears were analyzed (final magnification 200 times) and 100 sperm were evaluated per animal. Morphological abnormalities found in sperm were classified into two categories: a) head abnormalities: without characteristic curvature, in pinhead form, and isolated; b) tail abnormalities: folded, broken and isolated. Besides morphology, the presence or absence of cytoplasmic droplet in sperm was also evaluated.

### Evaluation of spermatogenic process and histopathological analysis

Aiming to evaluate the dynamic of the spermatogenic process, 100 transversal sections of seminiferous tubules per animal were analyzed at PND 40, 60 and 90, utilizing the method of attribution of values, according to the most mature germ cell present in the tubular epithelium [31]. The sections were evaluated randomly and classified by the most advanced cell type present in the epithelium: score 1- only spermatocytes; score 2- young spermatids with round nucleus (stages 1 to 8 of spermiogenesis); score 3- spermatids in maturation phase, with ovoid or elongated nuclei (stages 9 to 14 of spermiogenesis); score 4- spermatids in maturation phase, with elongated nuclei (stages 15 to 18 of spermiogenesis); score 5- mature spermatids (stage 19 of spermiogenesis) in small quantity; score 6- mature spermatids (stage 19 of spermiogenesis) in medium and maximum quantity. Finally, the number of tubules classified into each score of maturity was multiplied by its respective score, with the resultant values summed and then divided by 100, resulting in a "mean score". In adult animals (90 days), a more detailed evaluation of spermatogenesis was made in seminiferous tubules, classifying them into stages of I-VI, VII-VIII, IX-XIII and XIV, according to the number of generations of spermatids, presence of mature spermatids localized on the lumen border or secondary spermatocyte [32].

At PND 40, 60 and 90 the testes and epididymides of rats were examined under light microscope for analysis of the epithelial aspect, ductular lumen and interstitial tissue for identification of possible histopathological damage.

### Number of Sertoli cells per seminiferous tubule

To evaluate the possible effects of *in utero *and lactational growth restriction on the process of proliferation of Sertoli cells, all the nuclei of Sertoli cells were counted in histological sections of the testis of 90-days-old rats, in 20 seminiferous tubules per rat at stage VII of spermatogenesis, classified according to Leblond and Clermont [33]. The evaluations were accomplished in a blind assay (without knowledge of the group to which each animal may belong).

### Determination of plasma concentrations of total testosterone

After the decapitation of male rats at different ages, the blood was collected from the rupture of cervical vessels in a heparinized tube for determination of plasma testosterone concentrations. The plasma was obtained by centrifugation (2400 rpm, 20 min, 3.5°C) and frozen at -20°C until the moment of hormonal dosing. The concentrations of total plasma testosterone were determined in the Clinical Analysis Laboratory at the Botucatu Medical School, by the chemiluminescence methodology on the DPC-brand Immulit automatic equipment.

### Statistical evaluation of results

For analysis of percentages between the number of pregnant females and those that presented gestation to term, the Fisher exact test was utilized and the data were expressed as a percentage. For comparison of other parameters evaluated among the experimental groups, the Student's t test or Mann-Whitney test were utilized, depending on the normal and non-normal distribution of the data, respectively. Statistical analyses were accomplished by the program Instat (version 3.0; GraphPad, Inc., San Diego, CA, USA). Data were expressed as mean ± standard error of mean (SEM). The limit of statistical significance was considered p < 0.05.

## Results

### Evaluation of maternal results

The diabetic rats showed a significant increase in food and water intake when compared to control group (data not shown). The polyuria was observed due to the increase of humidity in the cage but this metabolic parameter was not measured. All rats that received STZ became diabetic (n = 57) and 55 of them had positive pregnancy. However, only 45.45% had termed pregnancy. The control group was initially composed of 17 female and all of them had positive and termed pregnancy (100%). As the litters with less than 8 pups or litters that were cannibalized were discharged, only 7 and 12 litters were used for diabetic and control group respectively.

Diabetic rats presented lower body weight gain during pregnancy (Table 1). The length of gestation was similar between the experimental groups (data not show). Blood glycemic levels were greater than 200 mg/dL in the diabetic rats, indicating severe diabetes. Normoglycemia was observed in the control rats (< 120 mg/dL) (Table 1).

**Table 1:** Body weight and blood glucose concentrations in diabetic and non-diabetic rats during pregnancy.

	*Non-diabetic group (n = 17)*	*Diabetic group (n = 55)*
*Body weight (g)*		
GD 0	218.64 ± 6.28	219.35 ± 3.07
GD 7	239.07 ± 6.61	240.06 ± 2.93
GD 14	271.91 ± 6.74	262.27 ± 3.87
GD 21	310.18 ± 7.71	288.40 ± 6.27
Maternal Gain (GD 21-GD 0)	91.54 ± 2.95	69.05 ± 4.56^a^
*Blood glucose concentrations (mg/dL)*		
GD 0	111.16 ± 3.64	476.86 ± 10.06^a^
GD 7	106.63 ± 3.50	468.71 ± 1.77^a^
GD 14	96.94 ± 2.75	525.48 ± 24.47^a^
GD 21	84.76 ± 3.19	561.32 ± 13.43^a^

Values are expressed as mean ± SEM.^a ^p < 0.0001. Mann-Whitney test. GD: gestational day.

### Evaluation of male offspring after birth

The mean body weight and glycemic levels at PND 3 were smaller in the OD compared to OC (Table 2). On the other hand, on PND10 the glycemic levels were similar between the groups (Table 2).

**Table 2:** Body weight and blood glucose concentrations on postnatal days 3 and 10.

	OC	OD
*Body weigth (g)*		
PND3	10.09 ± 0.98 (n = 12)	6.43 ± 0.87^a ^(n = 7)
*Blood glucose concentrations (mg/dL)*		
PND3	106.83 ± 2.00 (n = 12)	61.23 ± 5.00^a ^(n = 7)
PND10	140.46 ± 3.00 (n = 12)	124.94 ± 6.00 (n = 7)

Values expressed as mean ± SEM.^a ^p < 0.05. Student's t-test.

OC - litters from control rats; OD - litters from diabetic dams

Pups of diabetic dams showed a significant retardation in the time of testicular descent and preputial separation. These delays in the sexual development were accompanied by reduced body weights in the OD compared to the OC (Table 3).

**Table 3:** Ages and weights of the litters on the days of testicular descent and preputial separation.

	OC (12 litters)	OD (7 litters)
Testicular descent (days)	19.93 ± 0.19	22.89 ± 0.44^a^
Weight (g) of pups at the day of testicular descent	44.92 ± 1.12	23.07 ± 2.85^b^
Preputial separation (days)	42.83 ± 0.39	45.13 ± 0.36^a^
Weight (g) of pups at the day of preputial separation	150.73 ± 3.24	96.48 ± 7.62^a^

Values expressed as mean ± SEM.^a^p < 0.05,^b^p < 0.01. Mann-Whitney test. OC - litters from control rats; OD - litters from diabetic dams

### Reproductive parameters evaluated in different phases of male sexual development

The body weight of OD group was lower at all ages analyzed, although at 90 days of age the difference was not statistically significant (Table 4). Glycemic levels were similar between groups independent of age. The absolute weights of the testis (PND40 and 60), epididymis (PND40, 60 and 90), prostate (PND60 and 90), seminal vesicle (PND60), as well as the relative weight of the epididymis (PND60) of OD group were lower when compared to OC (Table 4).

**Table 4:** Body weight, glycemia, absolute and relative weights of the male reproductive organs of rats at 40, 60 and 90 days old.

	40 days		60 days		90 days	
	OC (n = 8)	OD (n = 6)	OC (n = 8)	OD (n = 6)	OC (n = 6)	OD (n = 6)
*Body weight (g)*	148.89 ± 4.40	102.90 ± 5.17^b^	269.14 ± 9.54	219.38 ± 10.45^a^	375.76 ± 6.70	314.48 ± 25.46
*Glycemia (mg/dL)*	132.25 ± 12.18	142.33 ± 11.40	109.00 ± 3.71	113.67 ± 2.94	103.00 ± 2.37	103.00 ± 0.39
*Testis (g)*	0.69 ± 63.78	0.44 ± 49.10^a^	1.50 ± 0.05	1.12 ± 0.05^b^	1.63 ± 0.15	1.39 ± 0.10
*(g/100 g of BW)*	0.46 ± 29.60	0.42 ± 35.08	0.56 ± 0.02	0.52 ± 0.04	0.44 ± 0.05	0.45 ± 0.02
*Epididymis (mg) *	74.51 ± 6.44	49.50 ± 4.30^b^	271.56 ± 7.08	172.73 ± 13.92^b^	532.92 ± .92	423.70 ± 28.32^b^
*(mg/100 g of BW)*	49.74 ± 2.96	48.15 ± 0.74	100.85 ± 5. 26	80.13 ± 7.95^a^	143.17 ± 7.18	135.64 ± 2.73
*Prostate (mg)*	51.60 ± 5.74	40.98 ± 2.58	167.30 ± 1.22	112.70 ± 9.06^a^	429.72 ± 9.25	278.88 ± 14.95^b^
*(mg/100 g of BW)*	34.43 ± 3.54	40.08 ± 2.65	60.37 ± 5.07	51.84 ± 4.46	115.04 ± 7.96	91.23 ± 7.34
*S. vesicle (mg)*	28.58 ± 5.51	20.88 ± 2.71	432.63 ± 4.36	260.82 ± 38.65^a^	1120.00 ± 0.08	853.00 ± 0.10
*(mg/100 g of BW)*	18.24 ± 3.03	20.41 ± 2.59	156.32 ± 3.58	123.76 ± 26.25	298.30 ± 0.02	275.25 ± 0.03

Values expressed as mean ± SEM.^a^p < 0.05^b^p < 0.01. Mann-Whitney test. BW- body weight. OC- offspring of control dams; OD- offspring of *diabetic dams*.

At PND60, the number of mature spermatids and the daily sperm production in the testes of OD group were significantly lower when compared to OC. These same parameters, when analyzed in relative terms, were similar between the experimental groups (Table 5). Sperm number in the caput-corpus of the epididymis and the sperm transit time through this epididymal portion were reduced in OD compared to OC. The same parameters in epididymal cauda were similar between the two groups (Table 5). At PND90, both daily sperm production and the number of spermatids in the testis, expressed in relative terms, were greater in OD compared to OC. In the epididymis, sperm number and transit time in caput-corpus region did not differ between the experimental groups, while the sperm transit time and the number of sperm in epididymal cauda was lower in the OD group (Table 5).

**Table 5:** Sperm counts in rats at 60 and 90 days-old.

	60 days		90 days	
	OC (n = 8)	OD (n = 6)	OC (n = 6)	OD (n = 6)
*Testis*				
Daily sperm production (× 10^6^)/testis	24.38 ± 0.77	17.05 ± 1.70^b^	38.08 ± 0.85	34.50 ± 1.81
Daily sperm production (× 10^6^)/g of testis	20.98 ± 0.45	20.85 ± 1.26	24.65 ± 0.69	29.52 ± 1.15^a^
Number of spermatids (× 10^6^)/testis	148.70 ± 4.70	104.00 ± 10.38^b^	232.27 ± 5.16	210.62 ± 11.13
Number of spermatids (× 10^6^)/g of testis	127.97 ± 2.72	127.16 ± 7.67	150.60 ± 0.30	180.23 ± 6.97^b^
*Caput/corpus epididymis*				
Sperm number (× 10^6^)	59.71 ± 6.71	25.77 ± 6.21^b^	112.63 ± 6.90	92.03 ± 7.40
Sperm number (× 10^6^)/g of epididymal region	401.33 ± 30.55	279.41 ± 47.69^a^	365.95 ± 13.88	386.60 ± 14.23
Sperm transit time (days)	2.04 ± 0.21	1.47 ± 0.67^a^	2.97 ± 0.17	2.65 ± 0.15
*Cauda epididymis*				
Sperm number (× 10^6^)	27.07 ± 5.68	10.66 ± 5.10	188.08 ± 5.34	137.80 ± 10.55^b^
Sperm number (× 10^6^)/g of epididymal region	353.56 ± 53.11	249.18 ± 97.29	1024.25 ± 0.28	840.37 ± 26.67^b^
Sperm transit time (days)	1.07 ± 0.20	0.54 ± 0.18	4.95 ± 0.15	4.09 ± 0.26^a^

Values expressed as mean ± SEM.^a^p < 0.05,^b^p < 0.01. Student's t-test. OC- offspring of control dams; OD- offspring of diabetic dams.

Sperm morphology was similar between the groups, with both presenting 89% normal sperm and 84% of sperm showing retention of the cytoplasmic droplet, in the inferior medial third of the flagellum.

Evaluation of the spermatogenic process at different moments of reproductive development did not indicate differences (p > 0.05) in the mean maturation score of the seminiferous epithelium (peripuberty: 2.60 ± 0.14, 2.30 ± 0.22; postpuberty: 3.91 ± 0.13, 3.74 ± 0.07; adulthood: 4.01 ± 0.03, 4.01 ± 0.07, OC and OD, respectively). In the same manner, in 90-day-old animals there were no differences in the spermatogenic process and in the number of Sertoli cells between the experimental groups (Table 6).

**Table 6:** Seminiferous epithelium cycle and mean number of Sertoli cells per seminiferous tubule of 90-day-old animals.

	OC (n = 5)	OD (n = 5)
*Stages of the germinal epithelium*		
I-VI	41.75 ± 0.75	39.75 ± 1.32
VII-VIII	28.75 ± 1.49	31.00 ± 1.15
IX-XIII	24.75 ± 0.48	24.5 ± 1.44
XIV	4.75 ± 0.85	4.75 ± 0.48
*Mean number of Sertoli cells*	14. 70 ± 0.15	14.33 ± 0.18

Values expressed as mean ± SEM. Student's t-test. OC- offspring of control dams; OD - offspring of diabetic dams.

**Figure 1:**
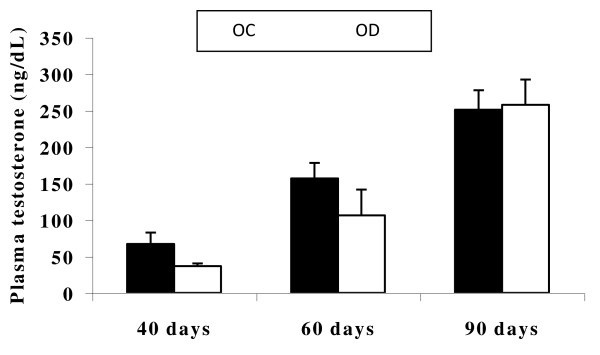
**Testosterone levels**. Plasma testosterone levels in the animals at the age 40 days (OC, n = 5; OD, n = 5), 60 days (OC, n = 7; OD, n = 6) and 90 days (OC, n = 11; OD, n = 8). OC: offspring of control dams; OD: offspring of diabetic dams. Values expressed as mean ± SEM. Student's t-test.

The qualitative histopathological analysis of the testes and epididymides did not reveal any alterations that could be attributed to the exposure to hyperglycemic environment during intrauterine development and lactation (data not shown). Plasma testosterone levels in the OD were similar (p > 0.05) to those of the OC in all studied ages (Figure 1).

## Discussion

In the present study, the classic pathophysiological signs of *Diabetes mellitus *such as hyperglycemia, polyphagia, polydipsia and polyuria, and lower body-weight gain during gestation were observed in the rats eight days after induction of diabetes, corroborating other studies in rodents [34-36].

The hypoglycemia of OD at PND3 may be explained as an exacerbated response of β-pancreatic cells of these pups due to the excessive hyperglycemic stimulus received in the intrauterine environment [37-39], although other factors may be involved. Low birth weight is the main characteristic of the IUGR and occurs frequently when high doses of STZ are used to induce diabetes [26,27,38]. Other studies have shown a reduction in body weight in fetuses of STZ induced-diabetic dams [34,40]. Data from literature demonstrate that intrauterine growth-restricted pups remain small up to adulthood [38], corroborating the results of the present study. Cross-fostering studies have shown that neonates of diabetic dams nursed by control rats recovered body weight around the weaning [27]. On the other hand, control pups suckled by diabetic dams presented a progressive delay in growth during neonatal life, with significant decrease in body weight from PND 14 [41]. Diabetic rats present decreased synthesis and milk ejection [42], and the milk from these animals has reduced levels of lactose, fat and protein [43], which apparently led to undernutrition in the offspring from nontreated diabetic dams. This factor during lactation period probably contributed with the persistent reduction in body weight in OD pups.

The increase in the ages of testicular descent and preputial separation in OD group may suggest that IUGR, besides impairing the normal growth and development of the male offspring, provokes retardation of the initial sexual development of these animals. Others studies corroborate our results showing that early malnutrition and consequent IUGR delay the onset of puberty in rats, associated or not with a body weight reduction [22,44,45]. Earlier reports have clearly shown that abnormal intrauterine environment affects the development of several fetal tissues and organs postnatally [46-48]. The present findings indicate impaired growth of male reproductive organs which may be explained, at least in part, due to the low body weight presented by these animals, since these same parameters, when analyzed in relation to body weight, were not significant.

The results of the histological analyses showed that the growth restriction caused by the maternal diabetes during gestation and lactation, did not impair the structural organization of the male offspring gonads, at any point during sexual development. At PND60 the reduction in testicular and epididymal sperm counts seem to be related to the smaller weight of these organs, since they were not significant when analyzed in relation to body weight. On the other hand, in adulthood the sperm reserves in the epididymal cauda were diminished independently of body weight reduction. Animals born from mothers exposed to protein restriction during gestation, another model of IUGR, showed a reduction in sperm reserves in adulthood, corroborating the result of the present study [49].

As a consequence of the decrease in sperm reserves in the cauda, the sperm transit time was accelerated in this epididymal region. The sperm transit time is a process regulated by androgens, which control the viscosity of epididymal luminal fluid and the contractility of the duct [50]. Other factors such as adrenergic, cholinergic, nonadrenergic noncholinergic innervation [51] and angiotensins, vasopressins and ocytocins in the blood [52] may act in the contractility of epididymal duct, interfering in the sperm transit. The accelerated transit of sperm through the epididymis promotes lower exposure of the gametes to the epididymal microenvironment which is crutial to the processes of sperm maturation [53,54]. Since plasma testosterone levels did not differ between male offspring of control and diabetic dams in the present study, the alteration of sperm transit time through the epididymis appears to have been independent of androgen action.

Recently, a clear relationship between low birth weight and adult renal disease has been stablished, probably related to the reduction of nephron endowment. Data in the literature suggests that individuals with IUGR have 70% greater risk of developing chronic kidney disease in adulthood [55,56]. Considering that epididymis and kidney structures have a common embrionary origin from Wolffian ducts [57] these results on renal diseases associated to IUGR may indicate a possible impairment on epididymal development and function at adulthood. Future studies are necessary to investigate the responsible mechanisms for this modification of sperm transit time through the epididymis and the possible consequences on sperm quality.

Nowadays, concern regarding intergenerational studies is well stablished, not only due to genetic factors but also to epigenetic mechanisms [58]. Burdge and co-authors [59] showed that alterations in the methylation status in specific genes of rat pups (F1) exposed to protein restriction during pregnancy can be transferred to subsequent generations, assuring the importance of epigenetic mechanisms. More investigations are necessary to evaluate the multigenerational effects of *in utero *and lactational exposure to a hyperglycemic environment.

## Conclusions

In conclusion, it is difficult to isolate the effects directly from diabetes and those from IUGR. Although the exposure to hyperglycemic environment during prenatal life and lactation delayed the onset of puberty in male rats, the IUGR, in the studied model, did not influenced the structural organization of the male gonads of the offspring at any point during sexual development. However the decrease in sperm reserves in epididymal cauda and the acceleration in sperm transit time in this portion of epididymis may lead to an impairment of sperm quality and fertility potential in these animals. Additional studies are needed in attempt to investigate the fertility of animals with intrauterine growth restriction by maternal diabetes and possible multigenerational effects.

## Competing interests

The authors declare that they have no competing interests.

## Authors' contributions

All authors participated in the design, interpretation of the studies, analysis of the data and review of the manuscript; EMPA, DCD, JEP, RS, CDBF and GTV conducted the experiments; EMPA and WDGK wrote the manuscript. This study represents EMPA's Masters Thesis presented to the State University of Campinas, under the advisory of WDGK. All authors read and approved the final manuscript.
